# A Novel Closed-Loop Control to Solve Light Source Power Fluctuations in the Fiber-Optic Gyroscope

**DOI:** 10.3390/s23104590

**Published:** 2023-05-09

**Authors:** Shijie Zheng, Mengyu Ren, Xin Luo, Hangyu Zhang, Guoying Feng

**Affiliations:** Institute of Laser & Micro/Nano Engineering, College of Electronics & Information Engineering, Sichuan University, Chengdu 610065, China

**Keywords:** power fluctuation, crosstalk, compensation, modulation and demodulation

## Abstract

The performance of a gyroscope is directly affected by the fluctuations in the light source power (LSP) in an interferometric fiber-optic gyroscope (IFOG). Therefore, it is important to compensate for fluctuations in the LSP. When the feedback phase generated by the step wave completely cancels the Sagnac phase in real-time, the error signal of the gyroscope is linearly related to the differential signal of the LSP, otherwise, the error signal of the gyroscope is uncertain. Herein, we present two compensation methods to compensate for the error of the gyroscope when the error is uncertain, which are double period modulation (DPM) and triple period modulation (TPM). Compared with the TPM, DPM has better performance, but it increases the requirements for the circuit. TPM has lower requirements for the circuit and is more suitable for small fiber- coil applications. The experimental results show that, when the frequency of the LSP fluctuation is relatively low (1 kHz and 2 kHz), DPM and TPM do not differ significantly in terms of performance; both of them can achieve an improvement of about 95% in bias stability. When the frequency of the LSP fluctuation is relatively high (4 kHz, 8 kHz and 16 kHz), DPM and TPM can achieve about 95% and 88% improvement in bias stability, respectively.

## 1. Introduction

After decades of development, the technology of IFOG has become very mature, and it is widely used in aviation, navigation, satellite navigation, and other fields [1,2]. In recent years, miniaturization and lowering costs have become hot topics of IFOG [3,4,5,6,7]. With the development of integrated chip technology, optical components of IFOG have gradually begun to be integrated into the chip, making miniaturization and low cost for IFOG possible. In 2011, GENER8 Co. and the Centre for Photonics (Ipswich, UK) integrated 24 optical components into a single chip [8]. In 2014, Bookham Co. integrated the light source, coupler, phase modulator and photodiode into a single chip, and its IFOG was successfully verified on a missile INS [9]. In 2017, a light source, three photodetectors, two phase modulators, and two couplers were integrated into a single chip by Tran et al. [10]. In 2019, KVH Co. integrated a polarizer and two Y waveguides into a single chip, its IFOGs achieving a bias stability of 0.048 °/h [11]. In 2022, Shang et al. produced an IFOG with a size of only 3 cm × 3 cm × 3 cm [12].

A superluminescent diode (SLD) is generally used in a low-cost IFOG, and the light source is generally packaged with an SLD, a thermoelectric cooler (TEC) and a thermistor. The package size is very large, and the driving circuit of the light source is also complicated. This is one of the reasons why there are almost no successful cases of miniaturized light sources for an IFOG, although Shang et al. realized the integration of a light source, photodetector and coupler in 2020. With the limitation of the volume of the TEC, the size after packaging still exceeded 26 mm [3]. In addition, a light source, three photodetectors, two phase modulators, and two couplers were integrated into single chip by Tran et al. in 2017, but the performance of these fiber-optic gyroscopes is relatively low, at just about 0.53 °/h [10]. Simplifying the light source of IFOG is an unavoidable step in the process of miniaturization and cost reduction, but simplifying the light source inevitably compromises its stability, which has a significant impact on the performance of the gyroscope [13,14,15].

There are two closed-loop control processes in a traditional IFOG, which are phase closed-loop control and 2π voltage closed-loop control [16,17]. Phase closed-loop control solves the problem of IFOG sensitivity reduction at high rotation rates, and 2π voltage closed-loop control solves the problem of the scale factor drift of the phase modulator. In this paper, we propose another closed-loop control to solve the problem of the fluctuation of LSP reducing the performance of the IFOG. Using our method, the compensation of the LSP fluctuation is realized without increasing the hardware cost. In the experiment, we modulate a variety of different waveforms, amplitudes, and frequencies for driving the current of the light source, and the experimental results prove that our method can reduce the impact of LSP fluctuations by about 95%.

## 2. Analysis and Simulation

### 2.1. Error Analysis

**Figure 1:**
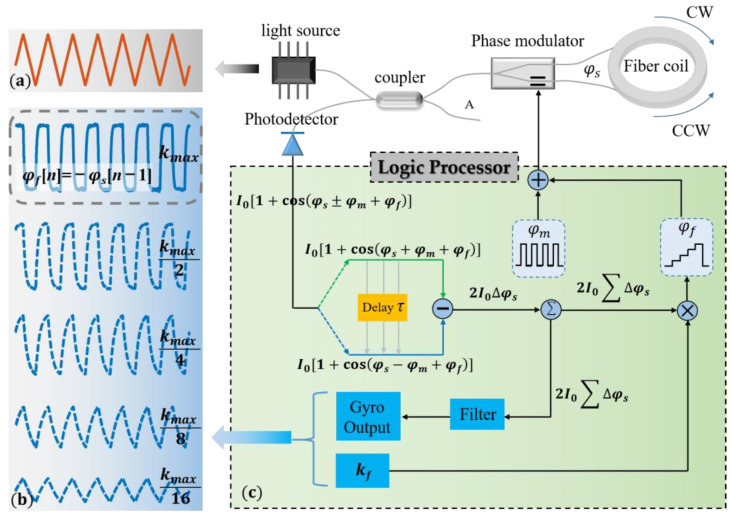
The system composition of IFOG, the signal of LSP and the signal of the output of IFOG. (**a**) Light source power signal. (**b**) The output signal of IFOG. (**c**) System components of an IFOG.

The IFOG system consists of a light source, coupler, phase modulator, fiber coil, photodetector and logic processor, as shown in Figure 1c.

There is a Sagnac phase *φ_s_* between the two beams of light rotating in opposite directions in the fiber coil, when the fiber coil is rotating. The Sagnac phase *φ_s_* is proportional to the rotation speed, resulting in a change in the intensity of the interferometric light. [18]. The Sagnac phase *φ_s_* can be expressed as:

(1) $$\varphi_{s}=\frac{2\pi LD\Omega_{\mathrm{in}}}{\lambda c}$$

where *L* is the length of the fiber coil, *D* is the diameter of the fiber coil, *Ω*_in_ is the rotation speed, *λ* is the average wavelength of light, and *c* is the speed of light in a vacuum. After closed-loop control of the gyroscope, if the total gain of the detection circuit is *G*, the output signal of IFOG can be expressed as:

(2) $$\Omega [n]=2GI_{0}\sum_{i=1}^{n}\Delta \varphi_{s}[i]$$

where *n* is a discrete signal sequence with a period of 2*τ*, Δ*φ_s_* is the increment of *φ_s_*, and *I*_0_ is proportional to LSP. Usually, we think that *I*_0_ is a constant. However, the LSP certainly fluctuates when the working environment of the gyroscope changes [19,20,21]. In addition, in the process of miniaturization and cost reduction, the light source and its drive circuit might be simplified, compromising the stability of the light source. Therefore, it is crucial to study the influence of LSP fluctuation on the gyroscope, and to compensate for them.

Due to LSP fluctuations, there will be a difference in the average light intensity between the first half period and the second half period, and this difference will be mistakenly thought by the IFOG system to be caused by the change in rotation speed. In the phase closed-loop control, the feedback phase *φ_f_* generated by the step wave will follow the rotation speed signal in real-time. After the closed-loop control phase, this difference in light intensity will eventually lead to an error in the output of the gyroscope.

The feedback phase *φ_f_* is linearly related to the height of the step wave. By integrating the light intensity difference between the first half period and the second half period, and then multiplying by a coefficient *k_f_*, we obtain a value that determines the height of the step wave, as shown in Figure 1c. The larger the coefficient of *k_f_*, the faster the response of the gyroscope. However, there is a maximum value *k_max_* which is a constant determined by the system, and when *k_f_* exceeds *k_max_*, the system is unstable.

When *k_f_* → *k_max_*, the feedback phase *φ_f_* generated by the step wave can almost completely cancel the light intensity difference of the previous period and, as a result, condition (*φ_f_*[*n*] = −*φ_s_*[*n* − 1]) is satisfied. According to our previous research work [22], if the differential signal of the light intensity, *I*_0_(*t*), is *β*(*t*), the output of the gyroscope can be expressed as follows [22]:

(3) $$\begin{array}{c} \Omega [n]=2G\sum_{i=1}^{n}I_{0}[n]\Delta \varphi_{s}[i]\\ +G\tau (\beta [n]-\beta [0]) \end{array}$$

where *τ* is the time required for light to pass through the fiber coil, and *I*_0_[*n*] is the average of light intensity, *I*_0_(*t*), in one period of the gyroscope. *β*[*n*] is the discrete signal of the differential signal of the LSP, which can be expressed as follows:

(4) $$\beta [n]=\frac{1}{2\tau }\int_{2n\tau -2\tau }^{2n\tau }\beta (t)dt$$

After considering the fluctuations in the LSP, the output signal of the gyroscope not only contains the desired Sagnac phase information, but also contains the undesired differential signal of the LSP. Subtracting a certain proportion of the LSP differential signal from the output signal of the gyroscope can effectively reduce the error caused by the fluctuation in LSP. However, this compensation method can only be used when condition (*k_f_* → *k_max_*) is satisfied.

The smaller the value of coefficient *k_f_*, the lower the system noise, and the higher the value of coefficient *k_f_*, the better the dynamic performance of the IFOG. That is to say, in practical applications, the coefficient *k_f_* is uncertain, and the prerequisite, (*k_f_* → *k_max_*), of the compensation method used in the previous research may not be satisfied [22].

When 0 < *k_f_* < *k_max_*, the crosstalk signal in the output signal of the gyroscope will no longer be the differential signal of the light source power. We consider an extreme case, when *k_f_* → 0, *φ_f_* hardly changes in a short time, and the output of the gyroscope can be expressed as:

(5) $$\begin{array}{cc} \Omega [n] & =G\sum_{i=1}^{n}\left(I_{B}[n]-I_{A}[n\right])\\ & =\frac{1}{2}G\sum_{i=1}^{n}\int_{2n\tau -2\tau }^{2n\tau }\beta (t)dt\\ & =\frac{1}{2}G[I(2n\tau )-I(0)] \end{array}$$

where *I_A_*[*n*] and *I_B_*[*n*] can be expressed as follows.

(6) $$I_{A}[n]=I_{0}(2n\tau -2\tau )+\frac{1}{2}\int_{2n\tau -2\tau }^{2n\tau -\tau }\beta (t)dt$$

(7) $$I_{B}[n]=I_{0}(2n\tau -2\tau )+\int_{2n\tau -2\tau }^{2n\tau -\tau }\beta (t)dt+\frac{1}{2}\int_{2n\tau -\tau }^{2n\tau }\beta (t)dt$$

When *k_f_* → *k_max_*, Equation (3) indicates that the crosstalk in the output of the gyroscope is LSP’s differential signal. However, Equation (5) shows that the crosstalk in the output of the gyroscope is the LSP signal rather than the LSP’s differential signal under the condition that *k_f_* → 0. That is to say, in the process of *k_f_* gradually decreasing from *k_max_*, the crosstalk in the output of the gyroscope will gradually change from the LSP’s differential signal to the LSP signal. For example, when the LSP is a triangle wave, in the process of *k_f_* gradually decreasing from *k_max_*, the crosstalk in the output of the gyroscope will gradually change from a square signal to a triangle signal, as shown in Figure 1a,b. Therefore, the impact of LSP fluctuations varies with coefficient *k_f_*. We have given the solution to the case of (*k_f_* → *k_max_*), in our previous research work [22]. In this paper, we present a compensation scheme for any *k_f_*, a significant advance on previous work.

### 2.2. Error Simulation

**Figure 2:**
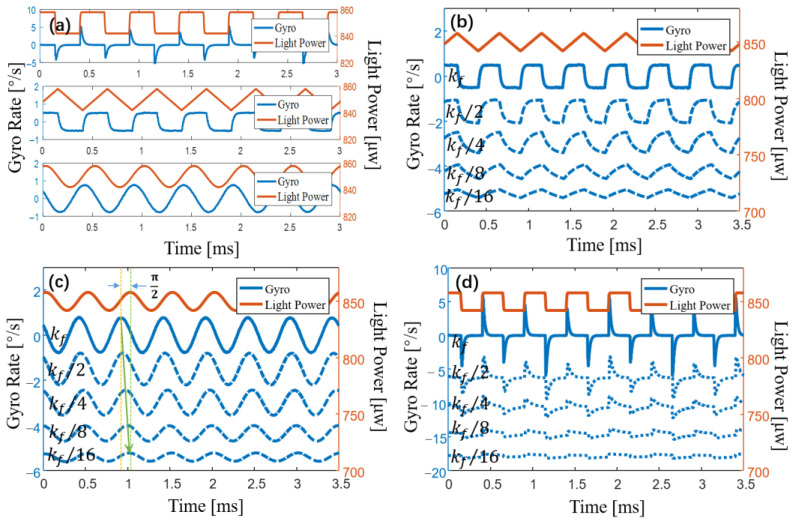
Simulation of crosstalk signal of the IFOG with different coefficients and waveforms. (**a**) When *k_f_* → *k_max_*, the output signal of the gyroscope is shown when the light source power is set to a square wave, triangle wave and sine wave, respectively. (**b**–**d**) As *k_f_* gradually decreases, the output signal of the gyroscope is shown when the light source power is set to a square wave, triangle wave, and sine wave, respectively.

We simulated the output signal of the IFOG with different LSPs and different coefficients of *k_f_*. When *k_f_* → *k_max_*, the LSP is set to a square wave, triangle wave, and sine wave, and the amplitude is 1% of the total power. The result is shown in Figure 2a.

When *k_f_* → *k_max_*, the simulation results are shown in Figure 2a, which are basically consistent with Equation (3). That is, the differential signal of the LSP is crosstalked into the output of the gyroscope. Spike pulses appear when the square wave jumps, which is in line with the results of our analysis. The differential signal of the triangle wave is a square wave, and a square wave also appears in the output of the IFOG. The differential signal of the sine wave has a π/2 phase difference from the original signal, and the simulation result is again consistent with our analysis.

When 0 < *k_f_* < *k_max_*, we simulate the output signal of the IFOG with the coefficient *k_f_* decreasing gradually, and the simulation results are shown in Figure 2b–d. With the decrease in the coefficient *k_f_*, the crosstalk signal in the gyroscope gradually changes from the differential signal of the LSP to the LSP signal. The simulation results are in line with the expected analysis results.

## 3. Principle of the Compensation Method

**Figure 3:**
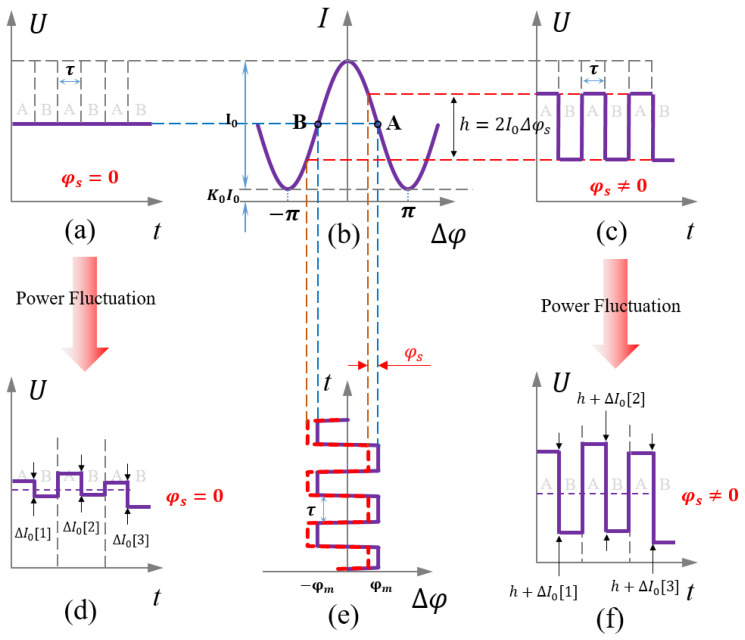
The light intensity of first half period and second half period. (**a**) The light intensity without rotation. (**b**) Response function of light intensity. (**c**) The light intensity with rotation. (**d**) After considering LSP fluctuation, the light intensity without rotation. (**e**) The modulated bias phase. (**f**) After considering LSP fluctuation, the light intensity with rotation.

When the LSP fluctuation is not considered, the corresponding light intensity with and without rotation is shown in Figure 3a,c, respectively. The output of the gyroscope can be obtained by integrating the difference between the light intensity of the first and the second half period.

After considering power fluctuations, the corresponding light intensity with and without rotation is shown in Figure 3d,f, respectively. Due to the LSP fluctuation, even if the rotation does not change, there will be a difference in the light intensity between the first and the second half period. In the first half period (0~*τ*), the interference light intensity is *I*_0_(1 − Δ*φ_s_*). In the second half period (*τ*~2*τ*), the interference light intensity is (*I*_0_ + Δ*I*_0_)(1 + Δ*φ_s_*). The difference in the interference light intensity between the first half period and the second half period is 2*I*_0_Δ*φ_s_* + Δ*I*_0_ + Δ*I*_0_Δ*φ_s_*. However, both Δ*I*_0_ and Δ*φ_s_* are very small, so the interference light intensity difference can be expressed as 2*I*_0_Δ*φ_s_* + Δ*I*_0_. Therefore, the difference in light intensity, Δ*I*_0_, caused by LSP fluctuation will be also integrated into the output of the gyroscope. Therefore, the key to compensating for the LSP fluctuations is calculating the Δ*I*_0_ in real-time.

We present two methods for the real-time demodulation of Δ*I*_0_, double period modulation (DPM) and triple period modulation (TPM). The two methods have their own advantages. The performance of DPM is better, but it is difficult to realize in a miniature fiber-optic gyroscope. Therefore, we present the method of TPM, which can be used in micro-optic fiber gyroscopes, but its performance is slightly worse.

### 3.1. Principle of DPM

**Figure 4:**
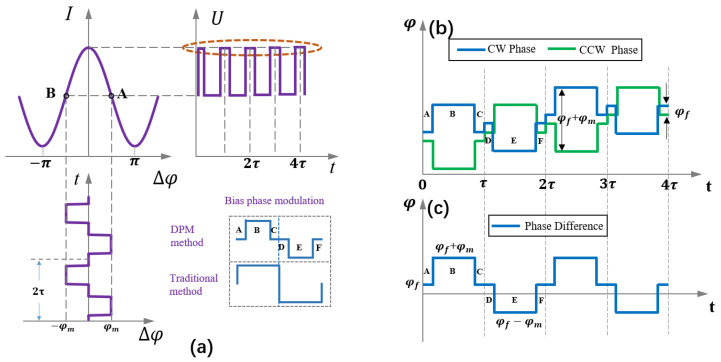
Light source power demodulation method of DPM. (**a**) Improved principle of square-wave bias modulation. (**b**) Modulation phase of CW and CCW light. (**c**) Phase difference between CW and CCW light.

In the traditional IFOG, a square wave is generally used to set the bias phase, and we divide the square wave modulation into six stages (A, B, C, D, E, and F), as shown in Figure 4a. In the B and E stages, we modulate the bias phase to ±*φ_m_*, but in the A, C, D, and F stages, we modulate the bias phase to zero. When the square wave and step wave are superimposed, the phase of the two beams rotating in opposite directions is shown in Figure 4c.

When *φ_m_* = π/2, the phase difference, *φ_smf_*, of the two beams rotating in opposite directions can be expressed as follows, at each stage.

(8) $$\varphi_{smf}=\left\{ \begin{array}{ll} \mathrm{AC}:\; & \Delta \varphi_{s}\\ \mathrm{DF}:\; & \Delta \varphi_{s}\\ B:\; & \Delta \varphi_{s}+\pi /2\\ E:\; & \Delta \varphi_{s}-\pi /2 \end{array}\right.$$

where Δ*φ_s_* → 0. Therefore, the light intensity at each stage can be expressed as follows.

(9) $$I=\left\{ \begin{array}{ll} \mathrm{AC}:\; & I_{0}(1+K_{0})\\ \mathrm{DF}:\; & I_{0}(1+K_{0})\\ B:\; & I_{0}(1+K_{0}-\Delta \varphi_{s})\\ E:\; & I_{0}(1+K_{0}+\Delta \varphi_{s}) \end{array}\right.$$

where *K*_0_ is caused by the incomplete cancellation of the two counter-rotating beams [23]. From Equation (8), we know that the phase difference between the two counter-rotating beams in stages A, C, D, and F is close to zero. However, the light intensity response is a cosine function, and the slope is zero at the origin. Therefore, the light intensity in stages A, C, D, and F is linearly related to the power of the light source. Finally, the average light intensity of the D and F stages minus the average light intensity of the A and C stages can reach Δ*I*_0_ = *I_DF_* − *I_AC_*.

### 3.2. Principle of TPM

**Figure 5:**
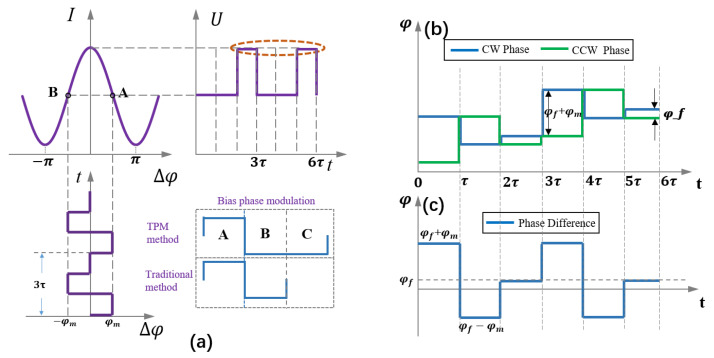
Light source power demodulation method of TPM. (**a**) Improved principle of square-wave bias modulation. (**b**) Modulation phase of CW and CCW light. (**c**) Phase difference between CW and CCW light.

In a miniature fiber-optic gyroscope, the length of the fiber coil is very short, only 100–200 m. The time it takes for the light to pass through the fiber coil is also very short, perhaps less than 1 μs. The DPM method requires the division of a period into several parts, which greatly increases the requirements for the circuit and may significantly affect the performance of the gyroscope [24]. Therefore, we use the method of TPM. The control period of the traditional fiber-optic gyroscope is 2*τ*, but the control period in TPM is 3*τ*. We add a period of *τ* to the traditional square wave modulation, and this period *τ* modulates the bias phase to zero, as shown in Figure 5a. When the square-wave bias phase and the step-wave feedback phase are superimposed, the modulation phases of clockwise (CW) and counterclockwise (CCW) light are shown in Figure 5b, and the phase difference is shown in Figure 5c.

When *φ_m_* = π/2, the phase difference *φ_smf_* of the two beams rotating in opposite directions can be expressed as follows, at each stage.

(10) $$\varphi_{smf}=\left\{ \begin{array}{l} A:\;\Delta \varphi_{s}+\pi /2\\ B:\;\Delta \varphi_{s}-\pi /2\\ C:\;\Delta \varphi_{s} \end{array}\right.$$

where Δ*φ_s_* → 0; therefore, the light intensity at each stage can be expressed as follows.

(11) $$I=\left\{ \begin{array}{l} A:\;I_{0}(1+K_{0}-\Delta \varphi_{s})\\ B:\;I_{0}(1+K_{0}+\Delta \varphi_{s})\\ C:\;I_{0}(1+K_{0}) \end{array}\right.$$

From Equation (10), we know that the phase difference between the two counter-rotating beams in the C stage is close to zero. However, the light intensity response is a cosine function, and slope is zero at the origin. Therefore, the light intensity in stage C is linearly related to the power of the light source. By calculating the light intensity difference between adjacent periods, we can obtain Δ*I*_0_ = (*I_C_*[*n*] − *I_C_*[*n* − 1])/3.

## 4. Experiment

### 4.1. Error Verification Experiment

**Figure 6:**
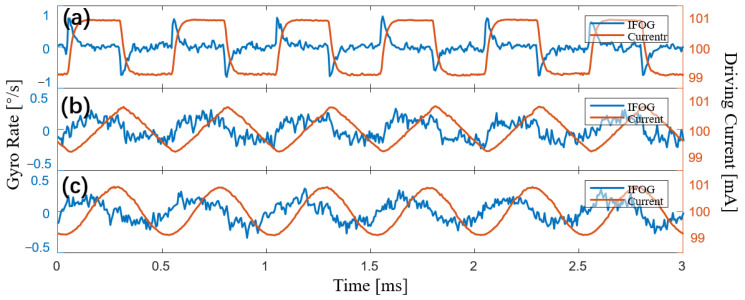
Experimental results of the output signal of the gyroscope and driving current of the light source. (**a**) Measurement results when the driving current is a square wave. (**b**) Measurement results when the driving current is a triangle wave. (**c**) Measurement results when the driving current is a sine wave.

When *k_f_* → *k_max_*, we modulate the driving current into a square wave, triangle wave, and sine wave, with a bias of 100 mA, a frequency of 2 kHz, and an amplitude of 1 mA. Figure 6 shows the output signal of the gyroscope and the modulated current signal. When the square wave of the driving current jumps, the gyroscope’s output signal displays a peak pulse. The square wave signal is superimposed on the output of the gyroscope when the driving current is a triangle wave. When the driving current is a sine wave, the output signal of the gyroscope displays a sine signal with a phase difference of π/2 from the drive current signal. All experimental results shown in Figure 6 agree with the simulation results shown in Figure 2a.

**Figure 7:**
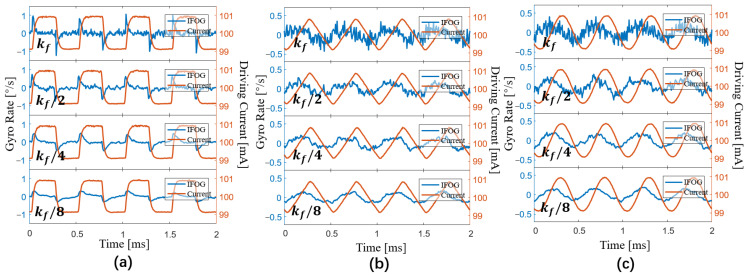
Experimental results of the output signal of the gyroscope when *k_f_* gradually decreases. (**a**) Results of the square-wave driving current. (**b**) Results of the triangle-wave driving current. (**c**) Results of the sine-wave driving current.

When 0 < *k_f_* < *k_max_*, the crosstalk signal in the gyroscope is no longer the differential signal of LSP. With a bias of 100 mA, a frequency of 2 kHz, and an amplitude of 1 mA, we modulate the driving current into a square wave, a triangle wave, and a sine wave, respectively. As shown in Figure 7, the output signal of the IFOG is measured as the coefficient *k_f_* decreases gradually. When the coefficient *k_f_* gradually decreases, the crosstalk signal in the output signal of the gyroscope eventually transforms from the differential signal of the LSP to the LSP signal itself. The experimental results, given in Figure 7, are consistent with the simulation results, presented in Figure 2b–d.

The two groups of experiments show that when the condition (*k_f_* → *k_max_*) is satisfied, the output signal of the gyroscope crosstalk the differential signal of the LSP. By subtracting a certain proportion of the differential signal of the LSP from the gyroscope’s output signal, it is able to effectively reduce the impact of LSP fluctuations [22]. However, this condition (*k_f_* → *k_max_*) is not satisfied in most practical applications, because the value of the coefficient *k_f_* is different in different applications. Thus, the compensation method of subtracting a certain proportion of the differential signal of the LSP from the gyroscope’s output signal fails to achieve the desired results.

We add the feature of compensating for the fluctuations of LSP to the closed-loop control of the traditional gyroscope. For this method, the value of the variable Δ*I*_0_ must be known in real-time, and we can demodulate Δ*I*_0_ using the DPM and TPM methods. The following sections detail the compensation results and the performance difference between DPM and TPM.

### 4.2. Light Source Power Compensation in Closed-Loop Control

The key to compensating LSP fluctuations is to acquire Δ*I*_0_. We present two methods for obtaining Δ*I*_0_: DPM and TPM. DPM’s frequency of demodulating Δ*I*_0_ is three times that of TPM, leading to better performance. Unfortunately, DPM must split a period into three parts, which significantly increases the circuit’s requirements. With a micro gyroscope, the length of the fiber coil is relatively short, and hence the period may be less than 1 μs, making it difficult to use the DPM method. As such, we propose the TPM method, which does not raise the circuit’s requirements.

**Figure 8:**
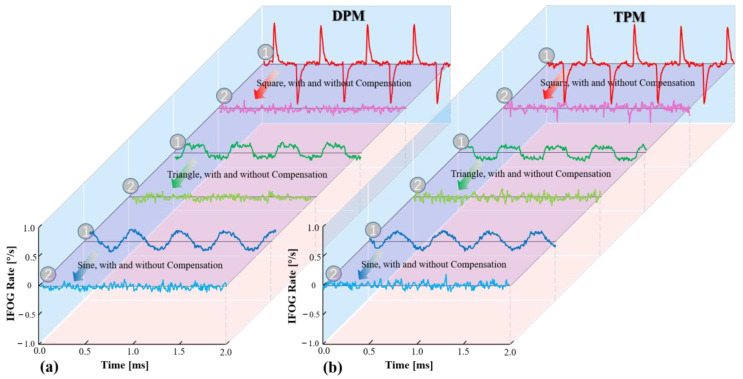
Experimental measurement results of the output signals of the gyroscope with and without compensation. (**a**) The result of the DPM method. (**b**) The result of the TPM method.

With a bias of 100 mA, a frequency of 2 kHz, and an amplitude of 2 mA, we modulate the driving current into a square wave, a triangle wave, and a sine wave in the experiment. The output signal of the gyroscope is then measured with and without compensation, as shown in Figure 8, where the curve labelled “1” represents the output signal without compensation and the curve labelled “2” represents the output signal with compensation.

**Figure 9:**
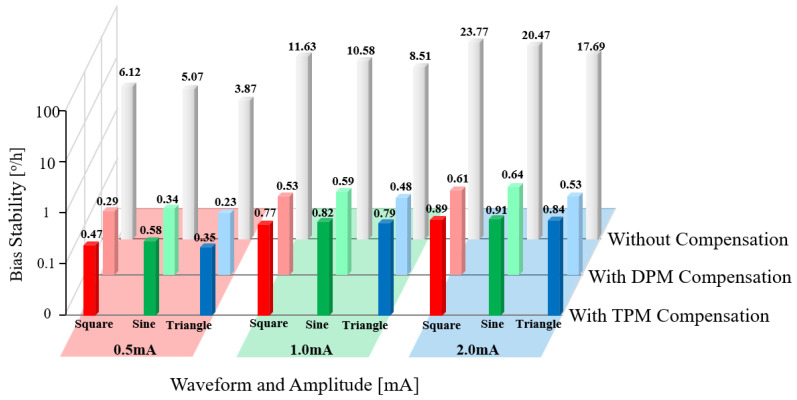
Experimentally measured bias stability of gyroscopes with different driving currents, and the bias stability of a gyroscope with the compensation of DPM and TPM.

Figure 8 shows that both the DPM and TPM methods are effective and that the mistake produced by the LSP fluctuations has been adequately compensated for. To further evaluate the performance difference between the two compensation methods, we modulated the driving current of the light source using various waveforms and amplitudes, as shown in Table 1. Figure 9 shows the results of measuring the bias stability of the gyroscope with and without DPM and TPM compensation.

When the light source is controlled by a constant temperature and current, the gyroscope’s bias stability is 0.089 °/h. The bias stability of the gyroscope degrades by two to three orders of magnitude when the driving current of the light source is intentionally modulated to create power fluctuations, as shown in Figure 9. In addition, for every 0.1 mA increase in the amplitude of the driving current, the bias stability of the gyroscope degrades by 8 to 13 times, compared to the case when the light source is controlled by a constant temperature and current. Both DPM and TPM can efficiently decrease the error caused by the fluctuations in the LSP. As shown in Table 2, we examined the variance in bias stability between DPM and TPM.

**Figure 10:**
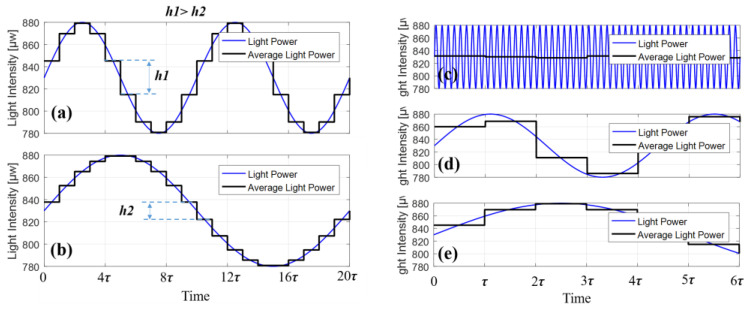
The difference in light intensity under different frequencies. (**a**,**b**) When the frequency is relatively low, the higher the frequency, the greater the error (*h*_1_ > *h*_2_). (**c**–**e**) When the frequency is too high and too low, the error becomes very small.

Figure 9 and Table 2 show that both DPM and TPM compensation methods increase bias stability by more than 90%. DMP’s higher improvement percentage, about 3–5%, is mostly attributable to its higher frequency for Δ*I*_0_ demodulation than that of TPM. When the frequency of LSP fluctuation is relatively low (as shown in Figure 10a,b), the higher the frequency, the greater the difference in light intensity between the first half period and the second half period. However, when the frequency of the LSP fluctuation is too high, the light intensity difference will be very small, as shown in Figure 10c–e. That is to say, power fluctuations in a certain frequency range have the greatest effect on the gyroscope, and this range depends on the length of the fiber coil.

**Figure 11:**
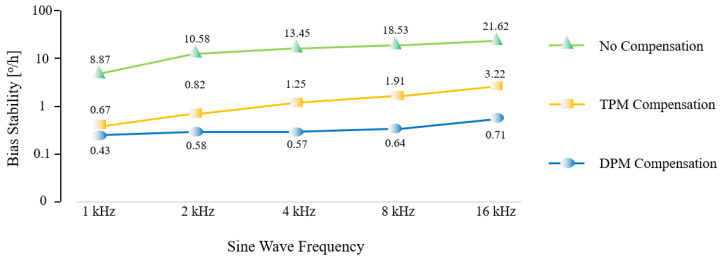
Experimentally measured bias stability of gyroscopes with different frequencies of light source power fluctuations, and the bias stability of gyroscopes with DPM and TPM compensation.

To further test the performance of the two compensation methods at different frequencies of LSP fluctuations, the driving current of the light source is modulated into a sine wave with an amplitude of 1 mA and frequencies of 1 kHz, 2 kHz, 4 kHz, 8 kHz, and 16 kHz. Figure 11 shows the results of a comparison of the bias stability of gyroscopes with DPM and TPM compensation at various frequencies of LSP fluctuations.

As shown in Figure 11, the bias stability of the gyroscope degrades as the frequency of the LSP fluctuations increases. Both the DPM and TPM methods are able to effectively reduce the detrimental effects of the LSP fluctuations, despite the varying frequency of the LSP fluctuations. When the frequency of the LSP fluctuations gradually rises, the performance of the DPM remains almost the same, but the performance of the TPM drops slightly; this is mostly due to the higher frequency of the Δ*I*_0_ demodulation of the DPM. The TPM demodulates Δ*I*_0_, based on the light intensity of adjacent periods, with a relatively low demodulation frequency. Thus, there is some performance reduction, but TPM can also achieve a bias stability improvement of over 80%. In actual applications, if the fiber coil is fairly long and there is sufficient time for light to pass through, we can pick the DPM compensation method. Unfortunately, with a small fiber coil, the time necessary for light to pass through the fiber coil is very short, making DPM not feasible. In this case, TPM can be used, since it also achieves an acceptable level of compensation.

## 5. Conclusions

The power fluctuations of the light source have a direct effect on the IFOG’s output signal, which has a significant effect on the IFOG’s performance. Nowadays, IFOG’s performance is close to its theoretical limit, and the miniaturization and low cost of IFOG have become hot topics of study. Throughout the miniaturization and cost-reduction processes, the light source may be integrated and simplified, which affects its stability and results in power fluctuations. We studied the effect of light source power fluctuations on the IFOG under various coefficients *k_f_*, and found that the effect of LSP fluctuations vary with *k_f_*. We present two methods of compensation, DPM and TPM, both of which can provide adequate compensation for any coefficient *k_f_*. Both DPM and TPM have achieved about 95% increase in bias stability, with DPM performing slightly better than TPM. Regrettably, DPM is difficult to achieve in the application of tiny fiber coils, but TPM can be applied in any gyroscope and has acceptable compensation performance. Our work increases the possibility of employing simpler light sources and driving circuits (e.g., no TEC, no constant temperatures control, and no constant current control) in IFOG, which has significant implications for ongoing attempts to miniaturize and lower the cost of fiber-optic gyroscopes.

## Figures and Tables

**Table 1:** Parameters of the drive current.

Waveform	Frequency	Amplitude
Square		0.5 mA
Triangle	2 kHz	1.0 mA
Sine		2.0 mA

**Table 2:** Bias stability of gyroscopes with different driving currents.

Waveform	Amplitude	Bias Stability without Compensation (°/h)	Bias Stability with DPM Compensation (°/h)	Bias Stability with TPM Compensation (°/h)
Square	0.5 mA	6.12	0.29 (↓95.3%)	0.47 (↓92.3%)
Square	1.0 mA	11.63	0.53 (↓95.4%)	0.77 (↓93.4%)
Square	2.0 mA	23.77	0.61 (↓97.4%)	0.89 (↓96.3%)
Sine	0.5 mA	5.07	0.34 (↓93.3%)	0.58 (↓88.6%)
Sine	1.0 mA	10.58	0.59 (↓94.4%)	0.82 (↓92.2%)
Sine	2.0 mA	20.47	0.64 (↓96.9%)	0.91 (↓95.6%)
Triangle	0.5 mA	3.87	0.23 (↓94.1%)	0.35 (↓91.0%)
Triangle	1.0 mA	8.51	0.48 (↓94.4%)	0.79 (↓90.7%)
Triangle	2.0 mA	17.69	0.53 (↓97.0%)	0.84 (↓95.3%)

## Data Availability

The data used to support the findings of this study are available from the corresponding author upon reasonable request.
